# THE JOYS OF GIVING: EXPLORING BIDIRECTIONAL RELATIONSHIPS BETWEEN ELDERS AND THEIR COMMUNITIES

**DOI:** 10.1093/geroni/igae098.1127

**Published:** 2024-12-31

**Authors:** Steffi Kim, Lena Thompson, Jordan Lewis

**Affiliations:** University of Alaska, Anchorage, Alaska, United States; University of Minnesota Duluth, Duluth, Minnesota, United States; University of Alaska Fairbanks, College of Indigenous Studies, Fairbanks, Alaska, United States

## Abstract

While Western community engagement with older adults research often focuses on topics such as community program involvement, volunteerism, and civic engagement, few studies have explored the role and impact of community engagement from an Alaska Native (AN) perspective. This presentation is part of a parent study consisting of 162 qualitative interviews with rural and 12 urban-based AN Elders over the course of 16 years and was analyzed using thematic analysis to explore the role of community in successful aging. AN Elders described bidirectional involvement in community engagement, in which they were involved as participants or as leaders. These engagements increased social connectivity and maintained cultural values. While few Elders described formal volunteer roles, they often supported one another through informal roles, like delivering food to others without access to traditional foods gathered from the land and the ocean. AN Elders also explained that the role of “Elder” is an important and respected community role, and often a title given based on their sharing of wisdom, experiences, and knowledge. A unique finding of this study is the bidirectional relationship between Elders and their communities. For example, Elders often expressed interest in participating in community activities, but communities also reach out to Elders to let them know they sought out their knowledge and experiences. Rural and urban based programs and services working to enhance engagement of Indigenous Elders in their community must consider expectations and desires communities may have of Elders and the desires of Elders to share their wisdom and experiences.

